# Circulating Tumor DNA Mutation Profiling by Targeted Next Generation Sequencing Provides Guidance for Personalized Treatments in Multiple Cancer Types

**DOI:** 10.1038/s41598-017-00520-1

**Published:** 2017-04-03

**Authors:** Yongqian Shu, Xue Wu, Xiaoling Tong, Xiaonan Wang, Zhili Chang, Yu Mao, Xiaofeng Chen, Jing Sun, Zhenxin Wang, Zhuan Hong, Liangjun Zhu, Chunrong Zhu, Jun Chen, Ying Liang, Huawu Shao, Yang W. Shao

**Affiliations:** 1 Jiangsu Province Hospital, The First Affiliated Hospital of Nanjing Medical University, Nanjing, Jiangsu China; 2 Geneseeq Technology Inc., Toronto, Ontario Canada; 3 Nanjing Geneseeq Technology Inc., Nanjing, Jiangsu China; 4 Department of Medical Oncology, The First Affiliated Hospital of Soochow University, Suzhou, Jiangsu China; 5 Jiangsu Institute of Cancer Research, Nanjing Medical University Affiliated Cancer Hospital, Jiangsu Cancer Hospital, Nanjing, Jiangsu China; 6 Department of Chemoradiotherapy, Yinzhou Hospital Affiliated to Medical School of Ningbo University, Ningbo, Zhejiang China; 7 Department of Medical Oncology, Sun Yat-sen University Cancer Centre, State Key Laboratory of Oncology in South China, Collaborative Innovation Centre for Cancer Medicine, Guangzhou, China; 8 Chengdu Institute of Biology, Chinese Academy of Sciences, Chengdu, Sichuan China

## Abstract

Cancer is a disease of complex genetic alterations, and comprehensive genetic diagnosis is beneficial to match each patient to appropriate therapy. However, acquisition of representative tumor samples is invasive and sometimes impossible. Circulating tumor DNA (ctDNA) is a promising tool to use as a non-invasive biomarker for cancer mutation profiling. Here we implemented targeted next generation sequencing (NGS) with a customized gene panel of 382 cancer-relevant genes on 605 ctDNA samples in multiple cancer types. Overall, tumor-specific mutations were identified in 87% of ctDNA samples, with mutation spectra highly concordant with their matched tumor tissues. 71% of patients had at least one clinically-actionable mutation, 76% of which have suggested drugs approved or in clinical trials. In particular, our study reveals a unique mutation spectrum in Chinese lung cancer patients which could be used to guide treatment decisions and monitor drug-resistant mutations. Taken together, our study demonstrated the feasibility of clinically-useful targeted NGS-based ctDNA mutation profiling to guide treatment decisions in cancer.

## Introduction

Cancers arise largely due to genetic mutations, yet are notorious for genetic diversity in different carriers^1, 2^. Personalized cancer treatment optimizes the clinical benefits for each patient by choosing targeted interventions based on that patient’s unique genetic profile and thus avoids ineffective therapies^3^. However, personalized treatment requires comprehensive and precise genetic profiling of the patient’s tumor. The development of next generation sequencing (NGS) has offered unprecedented progress in uncovering cancer genome characteristics and facilitating personalized cancer therapy due to its outstanding accuracy, sensitivity and high throughput^4–6^. Resected tumor tissues are frequently used in current NGS-based genetic testing^7^, but the operation is generally invasive, risky and often simply not possible, especially for cancer patients with advanced disease^8^. Additionally, cancer cells continuously acquire new mutations due to genomic instability and/or selective pressure from the tissue microenvironment and clinical treatment^9–11^. Thus, testing of a single tumor sample may overlook intra- and inter-tumor heterogeneity^12, 13^.

Circulating tumor DNA (ctDNA) is tumor-derived fragmented DNA circulating in blood along with cell free DNA (cfDNA) from other sources. ctDNA has an average length of 167 bp^14–16^. Although the mechanisms of ctDNA release into circulation have not yet been fully addressed, most reports consider apoptosis and/or necrosis of tumor cells as its main sources^16, 17^. ctDNA is therefore a genomic reservoir of different tumor clones and a good representation of tumor genomic diversity compared to a single tumor sample. Moreover, with a half-life from 16 minutes to a few hours^18–20^, ctDNA reflects the most up-to-date status of the tumor genome. Several methods have been developed to inspect tumor-specific mutations in ctDNA, such as allele-specific PCR, droplet digital PCR (ddPCR) and “BEAMing” (Beads, Emulsions, Amplification and Magnetics)^21–24^. Although these techniques are highly sensitive, their two major drawbacks are (1) low throughput on mutation scanning, and (2) the requisite of predefined knowledge of molecular targets to test for, meaning these techniques cannot be used for *de novo* mutation identification. Several groups have incorporated NGS into ctDNA mutation profiling and successfully identified numerous genetic alterations that correlate with disease progress and prognosis^25–28^. With growing interest in ctDNA testing, it is important to evaluate its utility in different types of solid tumors, not only as a diagnostic tool, but also as a tool for screening, monitoring and novel biomarker identification. In this study, we used a self-designed pan-cancer gene panel covering the exons of 382 genes for targeted NGS and established a clinically-applicable pipeline for ctDNA enrichment, sequencing and data analysis. This method was applied onto 605 patients with 29 different types of solid tumors. For comparison, tumor tissues from 344 patients were tested at the same time. Our data proves that mutation profiling of ctDNA by targeted NGS is feasible in clinical practice to guide treatment decisions during diagnosis and disease monitoring.

## Results

### Study Design and Patient Enrollment

**Figure 1:**
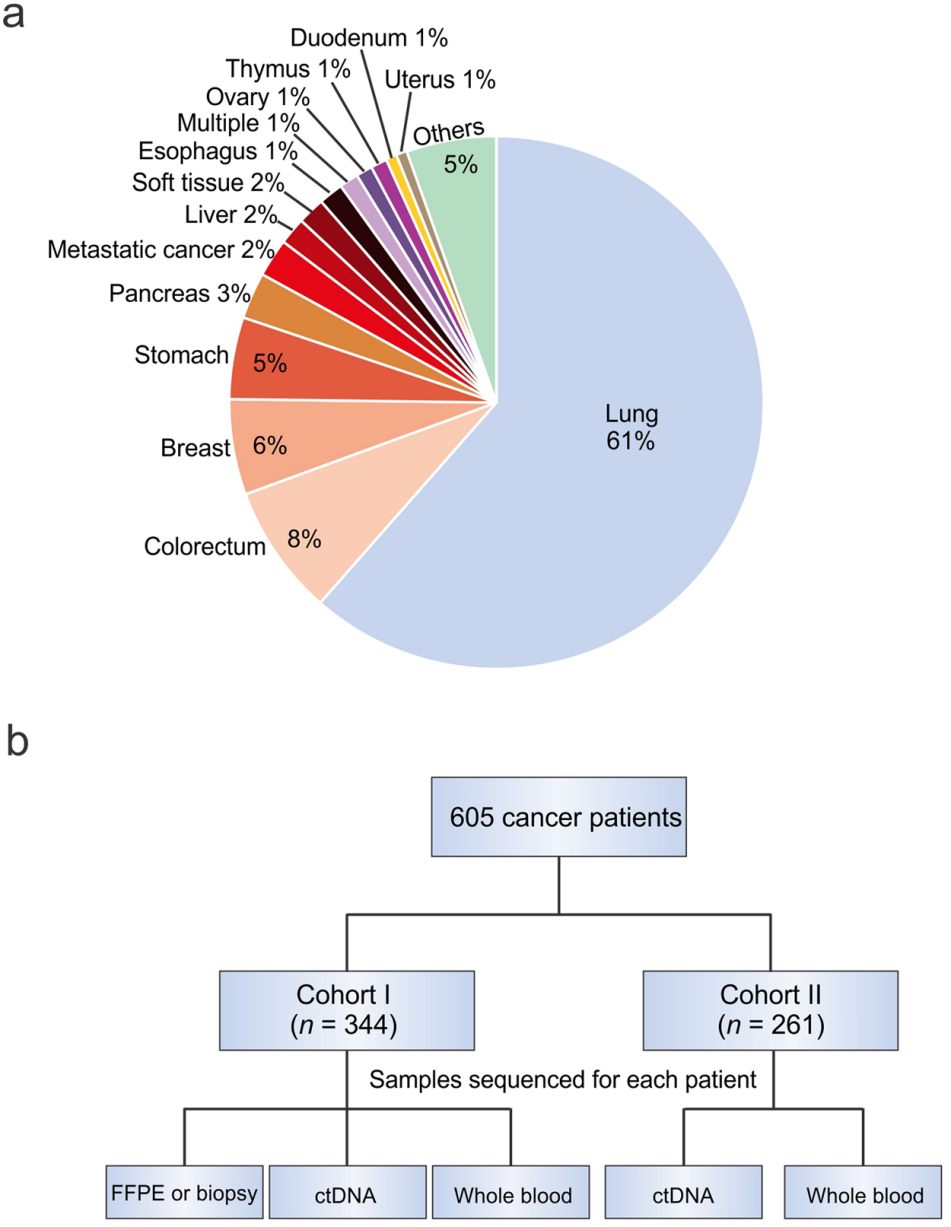
Study design and patient enrollment. (**a**) The percentage of different tumor types that enrolled in this study, including both Cohort I and II. Tumor types that were represented by less than 4 cases were classified as “Others”. (**b**) A schematic outlining our two-tiered study, including the cohorts and the specimens involved in this study.

605 cancer patients were randomly selected from 25 hospitals across Mainland China with a total of 29 types of tumors to ensure the representativeness and generalizability of this study. Brain tumors were excluded from this study due to the inhibition of release of ctDNA to blood by the blood-brain barrier^24^ (Supplementary Table 1 and Fig. 1a). Most patients had progressed to advanced cancer at the time of recruitment. Lung cancer (*n* = 373) was the largest category due to its high incidence in China^29^ and success in targeted therapy^30–32^. The other most common tumor types in our study were colorectal (*n* = 49), breast (*n* = 35) and stomach cancers (*n* = 30) (Fig. 1a). Two major criteria were applied for the exclusion of patients: 1) patients subjected to recent surgeries with primary tumors resected; and 2) patients currently receiving intensive targeted and/or non-targeted chemotherapies, with clinical imaging showing restrained tumor progression.

Patients were divided into two cohorts: one cohort (cohort I) had ctDNA samples collected along with their matched archived formalin-fixed paraffin-embedded (FFPE) or frozen tumor tissue samples, and another cohort (cohort II) only had ctDNA samples collected (Fig. 1b). Cohort I was designed to compare mutation profiles between ctDNA and tumor samples, as well as to assess the concordance rate for mutation detection between sample types. Cohort II was designed to compare ctDNA mutation profiles between two cohorts of patients. For each subject in both cohorts, genomic DNA from matched whole blood was also sequenced in order to discriminate of somatic and germline abnormalities.

### Targeted NGS-based Pan-Cancer Gene Mutation Profiling

**Figure 2:**
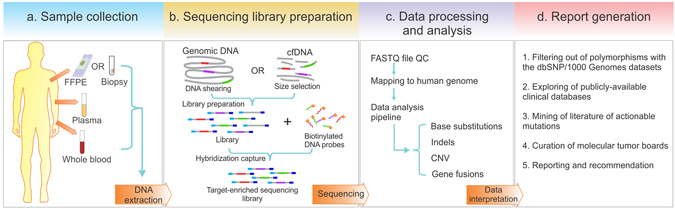
Workflow of targeted NGS-based mutation profiling. (**a**) Genomic DNA is extracted from multiple sample types. (**b**) Whole-genome libraries are prepared from fragmented genomic DNA or cfDNA, followed by hybridization capture with biotinylated DNA probes to establish target-enriched sequencing libraries for NGS. (**c**) Sequencing data undergoes quality control (QC), mapping and bioinformatic analysis to identify different classes of genomic aberrations. (**d**) Mutations identified are filtered and annotated according to related databases, and their clinical significances are interpreted in the final report.

Tumor tissue, cfDNA (which includes the ctDNA along with cfDNA from other sources) and whole blood controls were all sequenced using the same predefined panel (Fig. 2). Briefly, cfDNA was extracted from plasma, while genomic DNA was extracted from whole blood and either fixed or fresh tissue blocks (Fig. 2a). The median concentration of cfDNA from all 605 patients was 11.48 ng/ml plasma (Supplementary Fig. 1a). Extracted cfDNA was analyzed by the Agilent 2100 Bioanalyzer in order to detect genomic DNA contamination (Supplementary Fig. 1b) and subjected to an extra size-selection step using magnetic beads if contamination was found (data not shown). cfDNA and genomic DNA from all sample types underwent whole-genome library construction (Supplementary Fig. 1c and data not shown), followed by hybridization-based capture enrichment of 5,804 exons of 382 cancer-relevant genes and 37 introns of 16 genes frequently rearranged in solid tumors (Fig. 2b and Supplementary Table 2). Libraries after target enrichment were sequenced to high uniform depth on Illumina Miseq or HiSeq4000 platforms, depending on the sample type (see Methods). Sequencing data was analyzed using a customized bioinformatic pipeline optimized to accurately detect different classes of genomic alterations, including base substitutions, indels, copy number variations (CNV) and gene fusions (Fig. 2c). Finally, both germline and somatic genetic alterations in each patient were subject to manual data curation and reported (Fig. 2d). The average turnover time from receiving samples to reporting was 10 business days.

### High Concordance of Mutation Spectra between Matched ctDNA and Tumor Samples

**Figure 3:**
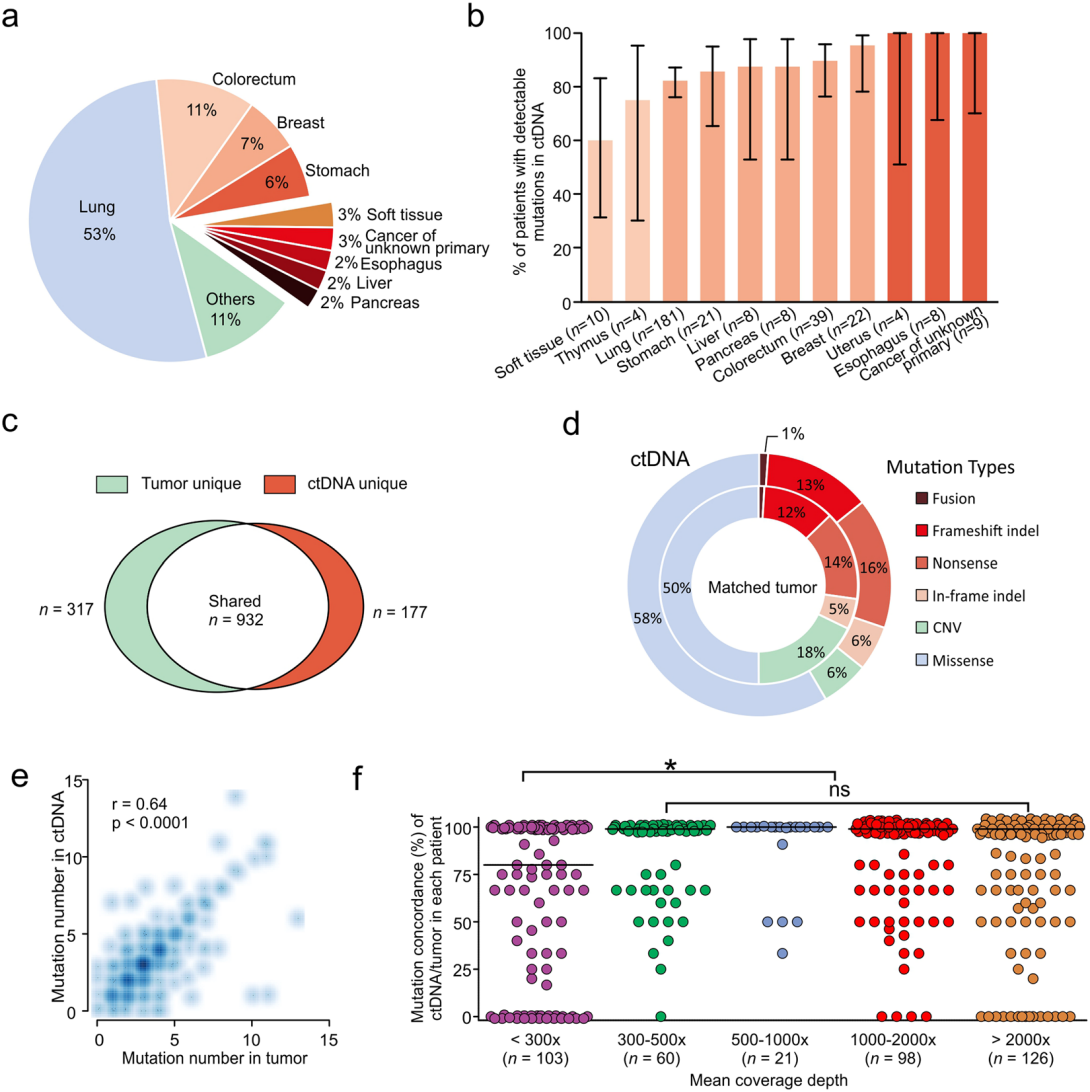
Mutation detection concordance between matched tumor and ctDNA samples in cohort I. (**a**) The composition of different tumors classified by their tissue origins. Tissue types that have less than 4 cases represented in the study are classified as “Others”. (**b**) The percentage of patients with mutations detected in ctDNA within different tumor types. Tumor types with less than 4 cases are not shown. (**c**) Shared and unique mutations identified in tumor and ctDNA samples. (**d**) The composition of mutation types in tumors and ctDNA. (**e**) Correlation of mutation numbers in ctDNA and matched tumors (Spearman’s rank test, p < 0.0001). The scatter dots were plotted according to mutation numbers identified per patient in ctDNA and matched tumors and the density represents the number of patients. (**f**) The correlation between mutation detection concordances in the matched tumor-ctDNA samples and sequencing coverage depth. The concordance rate was calculated by dividing the number of mutations in ctDNA to the number of mutations in matched tumor sample for each patient. Each dot represents one individual patient with median concordance rate shown by the black bar. *p < 0.05, Dunn’s multiple comparisons test; ns, not significant.

Cohort I included 344 patients representing 28 tumor types (Fig. 3a). ctDNA and tumor tissue blocks were sequenced simultaneously to provide a direct comparison of these two sample types. Patients with at least one somatic mutation identified in their ctDNA were defined as patients with detectable mutations in ctDNA, and were grouped by tumor types (Fig. 3b). Overall, ctDNA abnormalities were detected in around 80% of patients representing a majority of tumor types, with the exception of soft tissue tumors (mostly sarcoma) which showed the lowest rate of mutation detection in ctDNA.

In cohort I, a total of 1109 CNVs and mutations in ctDNA samples, and 1249 CNVs and mutations in tumors, were identified from 208 genes. 932 out of 1249 genetic abnormalities (74.6%) in tumor tissue were shared by matched ctDNA samples (Fig. 3c), although CNV was under-reppresented in ctDNA due to the current limitation of statistical approaches for CNV identification in NGS, especially in low tumor content samples^33^ (Fig. 3d and Supplementary Fig. 2a). Not surprisingly, CNV constituted 57% (181 out of 317) of the tumor-unique mutations. Therefore, CNVs were excluded in later analysis, and the concordance rate for all the other types of abnormalities increased to 87.0% (883 out of 1016). Meanwhile, ctDNA samples also harbor 177 unique abnormalities (including 24 CNVs) that were absent from tumor tissues, which may be ascribed to the inadequate representation of spatial and temporal heterogeneity by tumor tissue sequencing.

There is a significant correlation (Spearman r = 0.64, p < 0.0001) in the number of mutations identified between ctDNA and matched tumor tissue within each patient (Fig. 3e). 88% of tumor and 91% of plasma samples had 1–6 somatic mutations identified (median: 3 per sample for both). As expected, ctDNA, since it is mixed with cfDNA from other non-tumor sources, displayed significantly lower mutant allele frequencies (MAFs) with 66% of them below 10% (median: 5%), while mutations in tumor tissues had much higher MAFs with a median of 23% (Supplementary Fig. 2b).

Increasing sequencing coverage depth of ctDNA proved to be an efficient way to improve the detection sensitivity of tumor-specific mutations (Fig. 3f and Supplementary Fig. 2c). We chose 300× mean coverage depth of ctDNA as our cutoff for data analysis in this study. We could not detect any tumor-specific mutations in the ctDNA of 24% of patients using a mean coverage depth below 300× and a MAF detection cutoff of 1% (see Methods); however, when the coverage depth was increased to 300–500×, we observed a significant improvement in detection of matching mutations between ctDNA and tumor pairs. Further increasing the coverage depth to 500–1000×, 1000–2000× or >2000× did not significantly improve the matching rate (Fig. 3f). The number of mutations identified in tumor tissue and ctDNA, as well as their overlaps, are provided in Supplementary Table S3. Similar results were observed when comparing output of coverage depth variation between groups of different coverage depth in cohort I. When below 300× coverage depth for ctDNA sequencing, only 67% of tumor mutations were identified in ctDNA samples (Supplementary Fig. 2c). When coverage depth was increased to 300–500×, 88% of tumor mutations were detected in ctDNA, but the concordance rate was hardly improved when the coverage depth was increased up to 2000×. As a result, samples with coverage depth below 300× in this pilot group were excluded from other analyses.

There are several reasons to explain why mutations would be detected in tumor tissue but not in ctDNA, even at high coverage depth. One key reason is the low MAF of these mutations in tumor samples, suggesting extensive tumor heterogeneity in these patients (Supplementary Fig. 3a). Indeed, 43% of mutations that were undetected in ctDNA have a low MAF (below < 10%) in matched tumor tissues (Supplementary Fig. 3b). Other reasons include aged FFPE samples that may not represent the current mutation profile of the patient’s tumor, early stage cancers with low tumor burden, treatment intervention that may lower the possibility of detecting mutations in plasma and specific genetic regions that cannot be targeted well by NGS due to high GC-content or repetitive sequences that would influence ctDNA more than tissue sample due to the low MAF in ctDNA. Previous studies have showed that cfDNA concentration in plasma may be correlated with tumor burden and disease status^24^; however, we observed that the mutation detection rate was not significantly influenced by the plasma cfDNA concentration (Supplementary Fig. 3c).

### Targeted NGS-based Mutation Profiling of ctDNA Shows Great Potential for Clinical Practice to Guide Treatment Decisions

**Figure 4:**
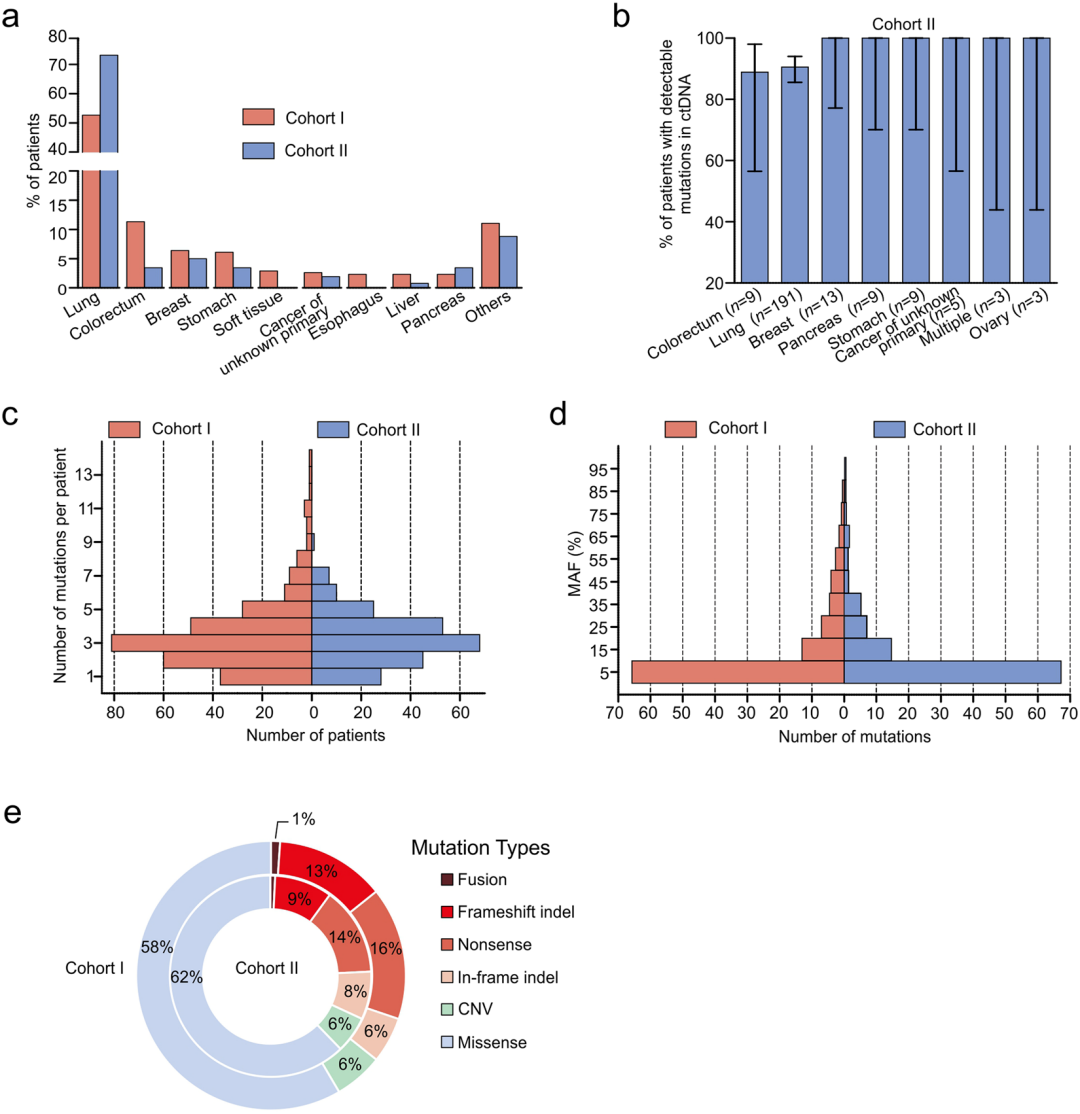
Similar ctDNA results between cohorts I and II. (**a**) There was a comparable distribution of tumor types covered in both cohorts I and II. (**b**) The fraction of patients with detectable ctDNA mutations in cohort II. (**c**) The distribution of mutation numbers identified per patient in cohorts I and II. (**d**) The distribution of MAFs in cohorts I and II. No significant difference was detected between the two cohorts in **c** and **d** by Mann-Whitney U test. (**e**) The distribution of different mutation types in cohorts I and II.

Our cohort II includes solely 261 ctDNA samples from patients with 28 types of solid tumors to validate the ctDNA mutation patterns observed in cohort I (Fig. 4). Promisingly, the results from cohorts I and II showed similar trends in several aspects, including percentage of patients with detectable mutations in ctDNA, number of mutations per patient, distributions of MAFs and different mutation types (Fig. 4b–e).

**Figure 5:**
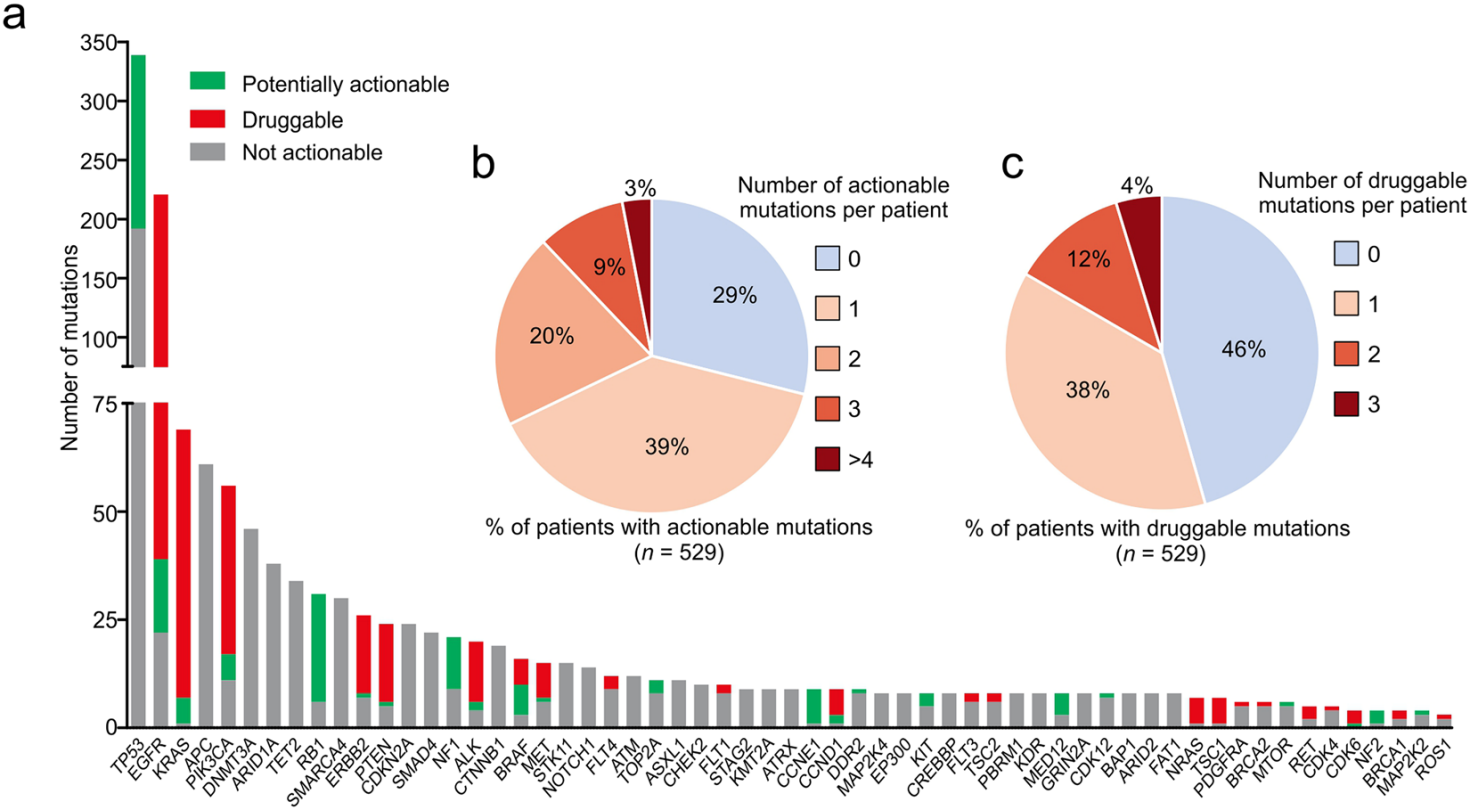
Clinically-actionable mutations identified in ctDNA samples. (**a**) Genes that are frequently mutated in cancer were ranked by their mutation frequency in all ctDNA samples tested, with the proportion of currently druggable mutations and potentially actionable mutations highlighted in red and green, respectively. Genes with low mutation occurrences (≤4) were not shown. (**b**) The percent of patients that presented with varying numbers of clinically-actionable mutations. (**c**) The percent of patients that presented with varying numbers of druggable mutations.

Combining ctDNA samples from cohorts I and II, somatic mutations were detected in 87% (529 out of 605) of patients. By compiling mutations identified in ctDNA samples of these two cohorts, it was observed that *TP53* (18.1% of all mutations), *APC* (3.3%) and *DNMT3A* (2.5%) were the most frequently mutated tumor suppressor genes, while *EGFR* (11.9%), *KRAS* (3.7%) and *PIK3CA* (3.0%) were the most frequently mutated oncogenes (Fig. 5a). 35.3% (662 out of total 1874 mutations in cohort I and II) of all mutations detected in ctDNA are potentially clinically-actionable, which are defined by three criteria: 1) they are related to FDA approved drugs or therapies; 2) they contribute to clinical therapy choice and outcome predictions in published clinical studies; and 3) they are targets of drugs or therapies that are currently under active clinical trials, showing promising intervention results^27, 34^ (Fig. 5a, green and red portions; Supplementary Table 4). Among these, 66.0% (437 out of 662) of mutations can be targeted by drugs already approved or currently in clinical trials (Fig. 5a, red portion). In summary, at least one clinically-actionable mutation was detected in 71% of patients (376 out of 529 patients with mutations detectable in their ctDNA, Fig. 5b), and 54% of patients had at least one druggable mutation (Fig. 5c). Overall, our data strongly suggests that this technique is an informative and effective approach to uncover druggable molecular targets, and has great potential to be used in guiding clinical treatment decisions.

### Characteristics of Mutations Identified in ctDNA from Chinese Lung Cancer Patients

**Figure 6:**
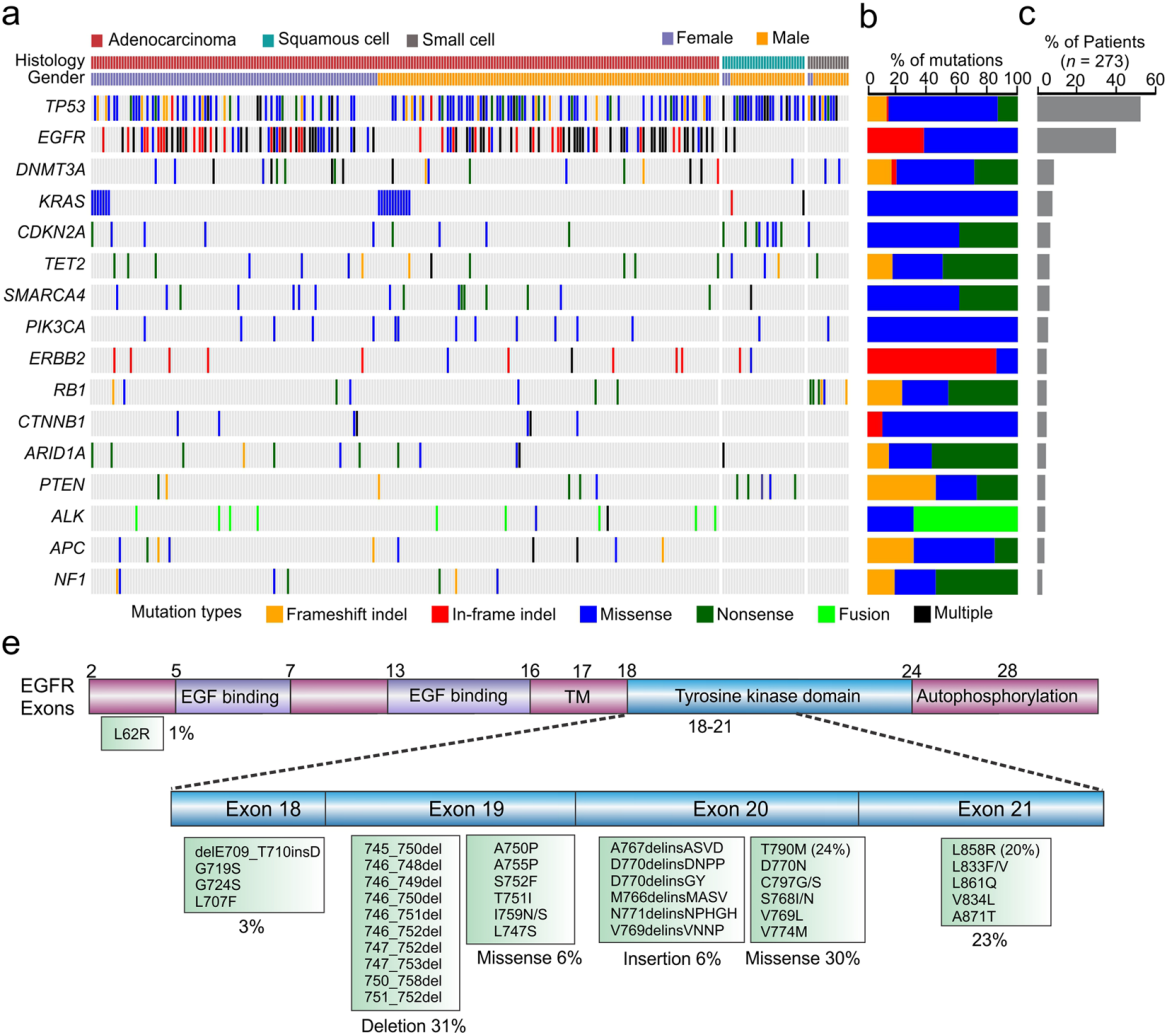
Mutation analysis of lung cancer patients. (**a**) A co-mutation plot of various types of mutations in the ctDNA of lung cancer patients. Only genes with more than 10 occurrences are shown in this plot. (**b**) The composition of mutation types within each gene. (**c**) The percentage of patients with mutations in each gene. (**d**) The specific mutations identified in EGFR and their frequencies in the ctDNA of our cohorts.

Our study also comprehensively analyzed the mutation spectrum in Chinese lung cancer patients. 273 lung cancer patients involved in this study were documented with clear clinical histological diagnosis and cancer stage classification. Adenocarcinoma represents 84% (*n* = 228) of all cases and no obvious gender difference in susceptibility to this subtype was observed (Fig. 6a). However, for squamous cell (*n* = 30) and small cell carcinoma (*n* = 15), more male patients were observed than females. By performing a co-mutation plot of mutations identified with the highest incidences in these patients, we showed that *TP53* mutations (52.38%) appear most frequently in ctDNA taken from patients with all types of lung cancers, followed by *EGFR* mutations (~40%) that exhibit strong preference to patients with adenocarcinoma (Fig. 6a and c). Different genes displayed variable preferences for different types of mutations. For example, *EGFR* is prone to adopt in-frame indel and missense mutations, while *ALK* is prone to forming fusion genes (Fig. 6b). In *EGFR*, the most prevalent mutations are exon19-deletion, T790M, L858R and exon20-insertion, with other types of activated *EGFR* mutations detected at lower frequencies (Fig. 6d). T790M mutations were only present in patients with resistance to tyrosine kinase inhibitor (TKI) treatment^35^. *EGFR*-C797G/S mutations, which were reported as the acquired resistance mutations in patients treated with the third generation of TKI, AZD9291^36^, were also detected.

It was observed that *KRAS* mutations are detected in 8% of patients, mainly those with adenocarcinoma, and are mutually exclusive from *EGFR* mutations as previously reported^37, 38^, although one patient harbored *KRAS*-G12L, *EGFR* amplification and exon19-deletion simultaneously (Fig. 6a). In squamous cell carcinoma, the occurrences of *CDKN2A* (27%) and *PTEN* (17%) mutations become more frequent compared to other cancer subtypes, while in small cell carcinoma, *RB1* mutations become more prevalent (40%) (Fig. 6a). Taken together, our study fully validates the use of targeted NGS-based genetic testing of ctDNA samples in clinical practice for guiding therapy decisions and monitoring treatment responses in lung cancer.

## Discussion

In this study, we presented that targeted NGS of ctDNA is non-invasive, can be completed with a fast turnover time, and can be standardized to generate reproducible data for routine clinical practice.

As we know, ctDNA is vigorously diluted by fragmented DNAs from normal cells, only taking up 0.01~10% of total cfDNA^39^. A critical advantage of targeted NGS compared to whole genome or exome sequencing is its increased coverage depth and sensitivity on genes of interest in a cost-effective manner for clinical practice. In this study, we designed a targeted pan-cancer gene panel, and identified mutations in all the patients with a median of 3 mutations per case. In cohort I, targeted NGS of ctDNA is equally effective to that of tumor tissues in identifying somatic mutations, with the exception of CNV detection, which needs to be further optimized for sensitivity by NGS. In addition to mutations shared with matched tumor DNA, ctDNA also harbored unique mutations that could be ascribed to spatial and temporal gaps between these two sample types. To validate the reproducibility of using ctDNA genetic testing as an independent test without matched tumors, we tested ctDNA alone in cohort II and observed similar number of mutations and mutation types as in cohort I.

In previous studies, a coverage depth as high as 10,000× has been reached in order to uncover rare ctDNA mutations^26^. However, high NGS coverage depth competes with sequencing errors to balance mutation identification with low false discovery rate^40^. Therefore, different detection thresholds should be set up for genetic testing during the diagnosis stage for *de novo* discoveries and during the disease monitoring stage for known mutations identified in primary tumors. To justify the minimum coverage depth enough to spot mutations in a cost-effective manner, we analyzed the mutation detection rate and concordance at different levels of coverage depth, and found that 300× mean coverage was the minimum requirement for mutation detection with the cutoff of MAF detection set at 1%, with increasing coverage depth leading to slightly increased mutation detection. In our study, most patients have progressed to advanced cancers when recruited. Therefore, with a MAF cutoff of 1%, we were able to detect at least one somatic mutation in 87% of patients. However, in most early-stage solid tumors or post-treatment patients, ctDNA levels are very low and thus need ultra-deep sequencing (>10000×) in order to detect rare mutations. Recently, accurate methods have been developed to reduce sequencing-related artifacts at these ultra-deep coverage depths^41, 42^. For example, Newman *et al*. developed a new method combining *in silico* elimination of stereotypical background artifacts with a molecular barcoding strategy for accurately identifying ultra-low frequency mutations in ctDNA samples. However, this technology will be costly for large gene panels in clinical practice, and is more suitable to be used for more defined, smaller gene panels.

A small discordance between mutation profiles from tissue and liquid biopsies is generally common. On one hand, a liquid biopsy harbors the mutations of all cancerous lesions (primary and metastases). Thus, ctDNA sequencing can detect mutations that cannot be identified in one single biopsy from tumor tissue. On the other hand, in cancers with low tumor burden, several mutations identified in tissue samples may not be detected in liquid biopsies due to the low content of ctDNA in cfDNA. Thus, when considering how to interpret mutations detected in both tissue and ctDNA samples during clinical management, we recommend combining all high-confidence somatic mutations present either in tissue or liquid biopsy samples for the generation of a final report. Regarding clinical reports, we adopted the strategy of involving multiple reviewers from molecular tumor boards to independently, then collaboratively, review mutation results. Our reporting strategy is relatively labor intensive and time-consuming, but the combined independent and collaborative reviews are not only important but also beneficial to the interpretation of clinically-actionable mutations.

The purpose of accessing the tumor genome is to acquire useful mutation information, guiding clinical therapeutic decisions. In our study, 71% of patients that have detectable mutations in their ctDNA have at least one clinically-actionable mutation with 76% having suggested drugs approved or in clinical trials. Targeted drugs are only effective when certain mutations are present. Drug-resistant mutations were also identified in our study, and identification of these will avoid unnecessary treatment. Although targeted agents for tumor suppressor genes are not currently available, such as *TP53*, *RB1* and *BRCA1/2*, their loss of function was shown to influence efficacy of some chemotherapy drugs^43–45^ and thus mutations in these genes are worthy of being taken into account when making therapeutic decisions. Further analysis of lung cancer, the most prevalent cancer type in our study cohorts, revealed a similar mutation distribution in the Chinese population as previously-reported^46, 47^. *EGFR* was mutated in 46.9% of adenocarcinoma cases, which could instruct the clinical application of EGFR TKI instantly. We also observed a much lower detection rate of KRAS (8%) in Chinese patients than the 33% reported by the Cancer Genome Atlas Research Network in 2014^38^. However, since the patients we tested have undergone various levels of chemotherapy or targeted treatments, their genomic profiles were likely reshaped by drug selection, restraining further interpretation of the results.

In summary, targeted NGS-based ctDNA mutation profiling is a non-invasive and sensitive tool to monitor tumor development, treatment response and drug resistance. With the flexibility to update customized targeted gene panels to adapt to different tumor types and to incorporate new discoveries, it offers a cost-effective platform as a routine clinical test to guide cancer treatment.

## Methods

### Patients and Sample Collection

Between November 2014 and October 2015, a total of 605 cancer patients were enrolled from hospitals across China (Supplementary Table 1). All patients were informed of sample collection and intended research usage. Written consent was collected according to ethical regulations of each participating hospital. The tests were performed in a centralized clinical testing center (Nanjing Geneseeq Technology Inc., Nanjing, China) according to protocols reviewed and approved by the ethical committee of Jiangsu Provincial Hospital. All methods were performed in accordance with the relevant guidelines and regulations.

5–10 ml peripheral blood was collected from each patient and placed into EDTA-coated tubes (BD Biosciences). Plasma was extracted within 2 hours of blood collection and shipped to the central testing laboratory within 48 hours. Formalin fixed paraffin embedded (FFPE) blocks/sections or fresh tumor tissues/biopsies were obtained from the hospitals, with confirmation by pathologists for diagnosis and tumor purity.

### DNA Extraction and Quantification

cfDNA was extracted using the NucleoSpin Plasma XS kit (Macherey Nagel) with optimized manufacturer’s protocols. Fresh tissue DNA and whole blood DNA were extracted using the DNeasy Blood & Tissue kit (Qiagen) according to the manufacturer’s protocols. FFPE samples were de-paraffinized with xylene and DNA was extracted using the QIAamp DNA FFPE Tissue Kit (Qiagen) according to the manufacturer’s protocols. Purified DNA was qualified by Nanodrop2000 (Thermo Fisher Scientific) and quantified by Qubit 2.0 using the dsDNA HS Assay Kit (Life Technologies) according to the manufacturer’s recommendations.

The size distribution of cfDNA was analyzed by the Agilent Technologies 2100 Bioanalyzer using the Agilent High Sensitivity DNA kit (Agilent Technologies) according to the manufacturer’s instructions. For cfDNA samples contaminated with genomic DNA, size selection was performed using Agencourt AMPure XP beads (Beckman Coulter) according to the manufacturer’s recommendations.

### Library Preparation

Sequencing libraries were prepared using the KAPA Hyper Prep kit (KAPA Biosystems) with an optimized manufacturer’s protocol. In brief, 1 μg of genomic DNA, which was sheared into 350 bp fragments using the Covaris M220 instrument (Covaris), or 2–100 ng of cfDNA, underwent end-repairing, A-tailing and ligation with indexed adapters sequentially, followed by size selection using Agencourt AMPure XP beads. Finally, libraries were amplified by PCR and purified for target enrichment.

### Hybridization Capture and Sequencing

Different libraries with unique indices were pooled together in desirable ratios for up to 2 μg of total library input. Human cot-1 DNA (Life Technologies) and xGen Universal blocking oligos (Integrated DNA Technologies) were added as blocking reagents. Customized xGen lockdown probes (Integrated DNA Technologies) targeting 382 cancer-relevant genes and 16 fusion genes were used for hybridization enrichment (Supplementary Table 2). The capture reaction was performed with the NimbleGen SeqCap EZ Hybridization and Wash Kit (Roche) and Dynabeads M-270 (Life Technologies) with optimized manufacturers’ protocols. Captured libraries were on-beads amplified with Illumina p5 (5′ AAT GAT ACG GCG ACC ACC GA 3′) and p7 primers (5′ CAA GCA GAA GAC GGC ATA CGA GAT 3′) in KAPA HiFi HotStart ReadyMix (KAPA Biosystems). The post-capture amplified library was purified by Agencourt AMPure XP beads and quantified by qPCR using the KAPA Library Quantification kit (KAPA Biosystems). Library fragment size was determined by the Agilent Technologies 2100 Bioanalyzer. The target-enriched library was then sequenced on Illumina MiSeq or HiSeq4000 NGS platforms (Illumina) according to the manufacturer’s instructions. For blood samples, sequencing depth was >100× mean coverage by non-PCR duplicate read pairs. For tumor specimens, sequencing depth was >300× mean coverage by non-PCR duplicate read pairs. For cfDNA samples, different sequencing depth was achieved for evaluation with the majority of the samples at >2000×. 99% of exons were at coverage >100× for all samples.

### Sequence Data Processing and Identification of Clinically-Actionable Mutations

Trimmomatic^48^ was used for FASTQ file quality control (QC). Leading/trailing low quality (quality reading below 15) or N bases were removed. Reads from each sample were mapped to the reference sequence hg19 (Human Genome version 19) using Burrows-Wheeler Aligner (BWA-mem, v0.7.12)^49^ with parameters (-t 8 -M). Local realignment around indels and base quality score recalibration were applied with the Genome Analysis Toolkit (GATK 3.4.0)^50^. GATK3.4.0 was applied to detect germline mutations from blood control samples. VarScan2^51^ was employed for detection of somatic mutations (somatic p-value = 0.1, minimum quality score = 15 and otherwise default parameters). Somatic variant calls presenting at less than 1% mutant allelic frequency in the paired blood control sample, but with at least 1% allelic frequency and at least 3 reads supporting variant alleles in tumor samples, were retained. We also filtered mutations reported in dbSNP (v137) and the 1000 Genomes database, but still kept mutations if they were also present in COSMIC database (v76). Annotation was performed using ANNOVAR^52^ using the hg19 reference genome and 2014 versions of standard databases and functional prediction programs.

Genomic fusions were identified by FACTERA^53^ with default parameters. In short, we set minimum number of breakpoint-spanning reads to 5, minimum number of discordant reads to 2 and minimum similarity required for alignment of read to fusion template to 95%. Copy number variations (CNVs) were detected using ADTEx (http://adtex.sourceforge.net) with default parameters. The main advantage of ADTEx is that it can derive absolute copy numbers without any a priori knowledge of levels of normal DNA contamination or ploidy of the tumor samples^54^. The algorithm takes not only depth of coverage (DOC) ratios but also allele frequency of germline heterozygous SNP (BAF) as inputs. The DOC ratios are smoothed by discrete wavelet transformation techniques prior to applying HMM to estimate polyploidy, normal contamination ratio and absolute CNVs. Germline CNVs from each patient were identified using the blood sample and normal human HapMap DNA sample NA18535 (Coriell Institute) for each captured region (exonic region). Somatic CNVs were identified using paired normal/tumor samples for each exon.

To facilitate implementation of genomically-informed therapy, we have adapted the established principles^55^ to create a three-tiered scale for levels of evidence to establish associations between genomic mutations and response to therapy. Level I data requires FDA-approved drugs or therapies (http://www.fda.gov/Drugs/). Level II data contributes to clinical therapy choice and outcome predictions in published clinical studies. We carried out literature searches to identify published prospective clinical studies pertaining to genomic alterations and their association with clinical benefits. For example, one such study might have been a case-control study demonstrating a statistically-significant association of a mutation with clinical benefit. Level III data requires drugs or therapies that are currently under active clinical trials (http://clinicaltrials.gov/), showing promising intervention results. All genomic alterations that can be targeted by drugs from Level I or Level III data were defined as druggable mutations.

At least two reviewers from the molecular tumor board independently compiled lists of candidate reportable mutations and manually reviewed the mutation calls based on various criteria: (i) not present in a segmental duplication region or a region with mapping score < 2; (ii) at least two distinct reads with different mapping positions (reads with the same mapping positions are more likely duplicates) supporting variant alleles; (iii) reads supporting variant alleles should be located on both strands; (iv) variant nucleotides should not always be located at the 3′ end of supporting reads; and (v) indel sequences should not be followed by near-identical closely located repeats. These independent lists were then combined by the medical director and returned to the board for group assessment. Once the molecular tumor board agreed on a final list of mutations to report, the medical director composed a formal medical report describing the mutations detected and the clinical action associated with each mutation.

### Data Deposition Statement

Sequence data has been deposited at the European Genome-phenome Archive (EGA, http://www.ebi.ac.uk/ega/), which is hosted by the EBI, under accession number EGAS00001002251.

## Electronic supplementary material

Supplementary Information
