# Supplementary material for: Circulating Tumor DNA Mutation Profiling by Targeted Next Generation Sequencing Provides Guidance for Personalized Treatments in Multiple Cancer Types
Source: Sci Rep. 2017 Apr 3;7:583. doi: 10.1038/s41598-017-00520-1 (PMC5428730; doi:10.1038/s41598-017-00520-1)
Supplement: Supplementary file 1 — Supplementary Information [file 41598_2017_520_MOESM1_ESM.pdf]

Supplementary information

Circulating Tumor DNA Mutation Profiling by Targeted Next Generation Sequencing Provides Guidance for Personalized Treatments in Multiple Cancer Types

Yongqian Shu[1,9], Xue Wu[2,9], Xiaoling Tong[2], Xiaonan Wang[3], Zhili Chang[3], Yu Mao[3], Xiaofeng Chen[1], Jing Sun[1], Zhenxin Wang[4], Zhuan Hong[5], Liangjun Zhu[5], Chunrong Zhu[4], Jun Chen[6], Ying Liang[7], Huawu Shao[3, 8], and Yang W Shao[2, *]

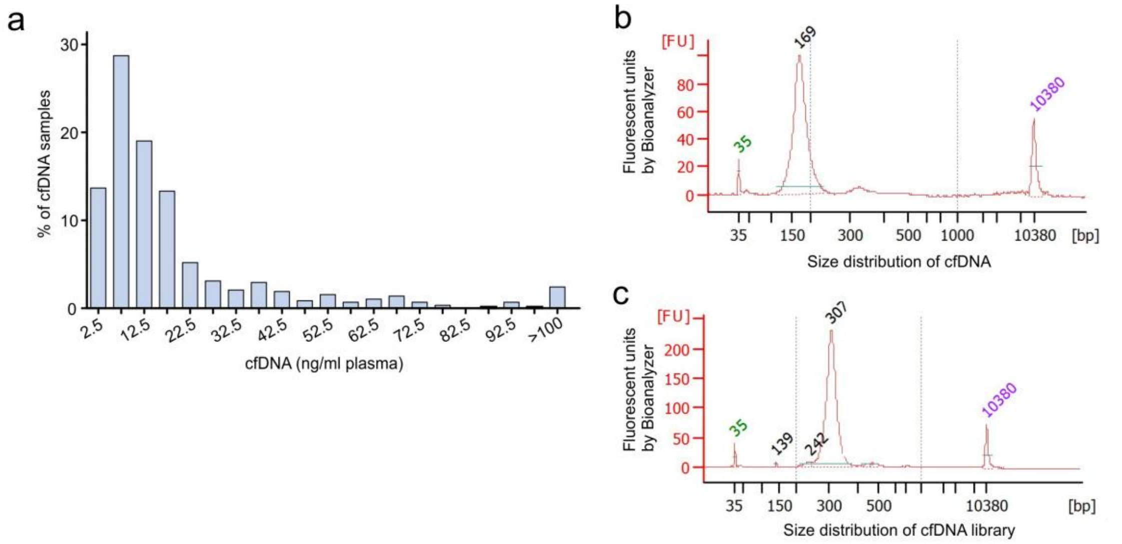

Supplementary Figure 1. Quantification of cfDNA and quality control of the cfDNA library. **a.** Distribution of cfDNA concentration in 605 plasma samples. **b.** Bioanalyzer analysis showing the size distribution of a high quality cfDNA sample. No large-sized genomic DNA contamination was detected in this sample. **c.** Bioanalyzer analysis showing the size distribution of a cfDNA library.

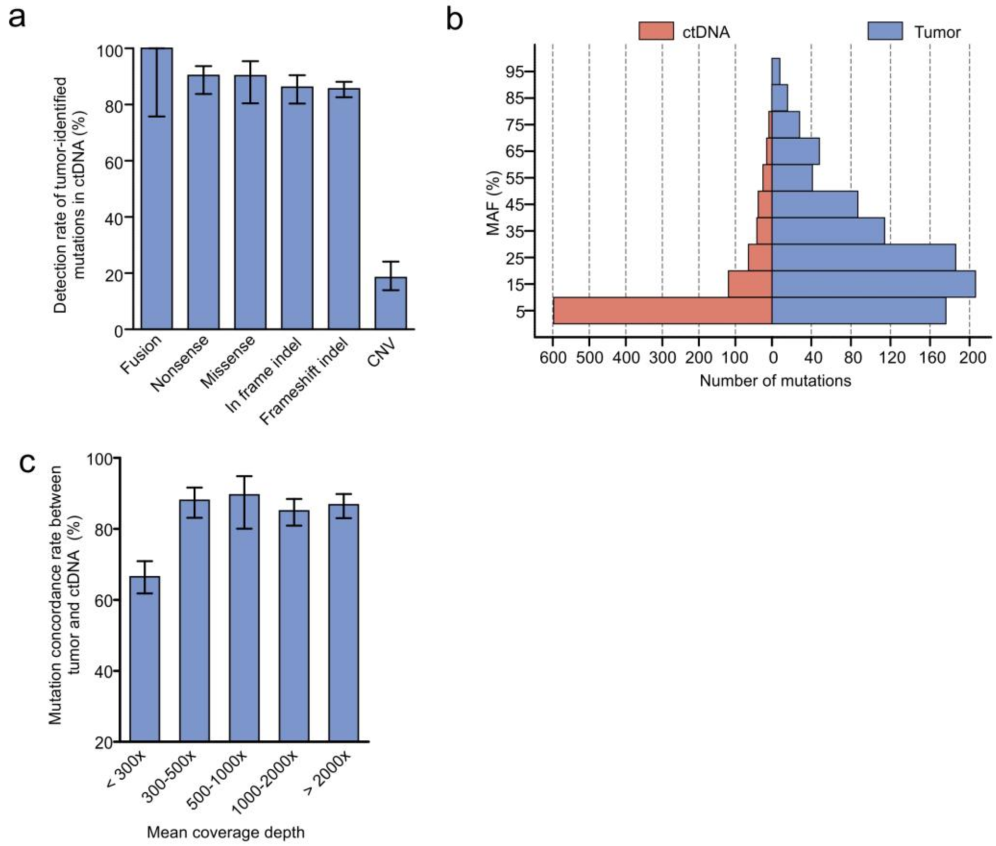

Supplementary Figure 2. Mutation analysis of matched tumor-ctDNA samples in cohort I. **a. The detection rate of tumor mutations in ctDNA across a variety of mutation types.** For each mutation type, the detection rate was calculated as the percentage of tumor mutations that were also detected in ctDNA. **b.** The distribution of mutant allele frequencies (MAFs) in ctDNA and matched tumors ($p<0.0001$, Mann-Whitney U test). **c.** Mutation concordance rate of tumor-identified mutations in ctDNA (i.e. the number of matched ctDNA-tumor mutations divided by the total number of mutations identified in the tumor tissue sample) per patient under different sequencing coverage depths. For **a** and **c**, error bars indicate the 95% Wilson confidence interval of calculated concordance rate.

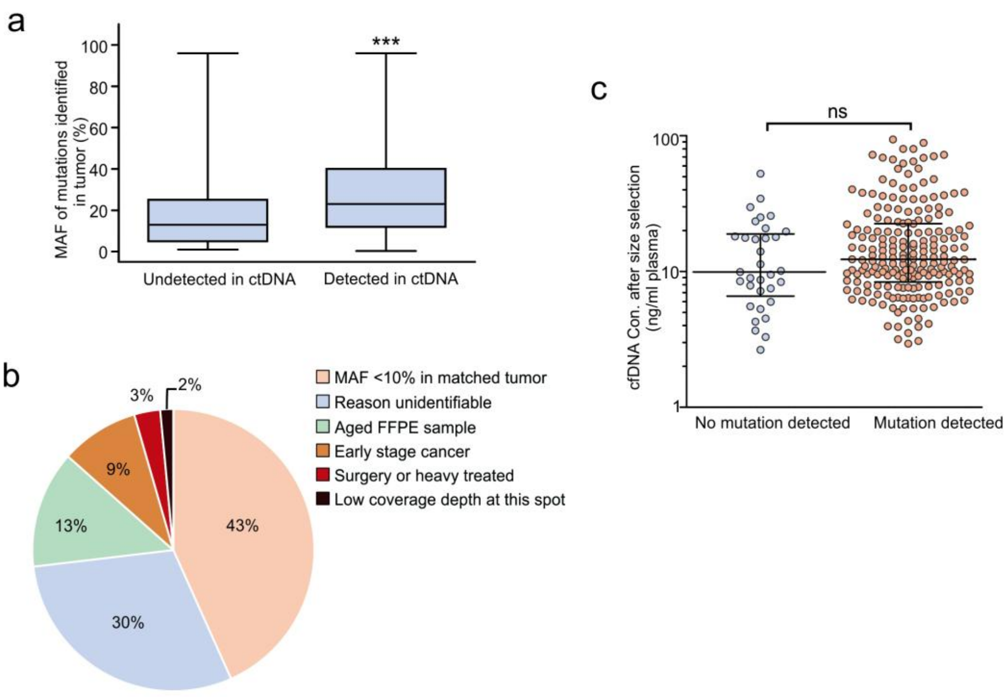

Supplementary Figure 3. Potential reasons to explain why some tumor mutations were not detected in matched ctDNA. **a.** Tumor mutations that were not detected in matched ctDNA have significantly lower MAFs than those detected in ctDNA. ***: $p < 0.001$, Mann-Whitney U test. **b. The proportion of mismatched mutations that could be explained by a variety of reasons. c.** There is no significant difference in cfDNA concentrations when cfDNA is divided into groups where mutations were detected or undetected. ns: not significant, Mann-Whitney U test.

Supplementary Table 1. Clinical characteristics of patients

Age at ctDNA test (*n* = 605)	Years
Median	60
25th– 75th percentiles	51-67
Range	18 - 89
**Gender**	***n***
Female	247
Male	358
**Cancer types**	***n***
Lung	372
Colorectum	48
Breast	35
Stomach	30
Pancreas	17
Cancer of unknown primary*	14
Liver	10
Soft tissue	10
Esophagus	9
Diagnosed with multiple cancer types**	7
Ovary	6
Thymus	6
Duodenum	4
Uterus	4
Gallbladder	3
Kidney	3
Parotid gland	3
Thyroid	3
Urinary tract	3
Skin	3
Adrenal cortex	2
Bile duct	2
Osteosarcoma	2
Prostate	2
Cervix	1
Nasal cavity	1
Nasopharynx	1
Peritoneum	1
Rectum	1
Scrotum	1
Small intestine	1

Clinical stage	*n*
I	1
II ~ III	63
IV	498
ND***	43
**Specimens**	*n*
Matched tumor-plasma pairs (training)	344
Plasma only (validation)	261
**Tumor tissue specimens**	*n = 344*
FFPE	264
Fresh	52
Pleural effusion and ascites	28

*Metastatic cancer with unknown primary site

**Patients with more than one type of cancer diagnosed simultaneously or sequentially

***Cancer stages were not determined

Supplementary Table 2. Genes covered by targeted NGS panel

ABCC2	BCL2L1	CDKN2A	DLG2	FANCF	HBA1	KDM5A	MSH6	PDGFRA	RAD51	SMC1A	TOP2A	ALK
ACTB	BLM	CDKN2B	DMNT3	FANCG	HBA2	KDR	MTHFR	PDGFRB	RAF1	SMC3	TP53	BCL2
ADH1B	BMPR1	CDKN2C	DNM2	FANCL	HBB	KIT	MTOR	PDK1	RARA	SMO	TP63	BCR
AIP	BRAF	CEBPA	DOT1L	FAT1	HDAC1	KMT2B	MUTYH	PHF6	RASGEF1	SOX2	TPMT	BIRC3
AKT1	BRCA1	CEP57	DPYD	FBXO11	HDAC2	KMT2C	MYC	PHOX2B	RB1	SPOP	TRAF2	BRAF
AKT2	BRCA2	CHD4	DUSP2	FCGR2B	HDAC4	KRAS	MYCL1	PICK3R1	RECQL4	SRC	TRAF3	ETV1
AKT3	BRD4	CHEK1	EBF1	FGF19	HDAC7	LEF1	MYCN	PIK3C3	RELN	SRSF2	TRAF5	ETV5
ALDH2	BRIP1	CHEK2	ECT2L	FGFR1	HGF	LMO1	MYD88	PIK3CA	RET	STAG2	TSC1	EWSR1
ALK	BTG2	CKS1B	EED	FGFR2	HNF1A	LYN	NBN	PIK3CD	RHOA	STAT3	TSC2	KMT2A
AMER1	BTK	CREBBP	EGFR	FGFR3	HNF1B	LYST	NCSTN	PIK3R1	RICTOR	STAT5A	TSHR	MYC
AP3B1	BTLA	CRKL	EGR1	FGFR4	HRAS	LZTR1	NF1	PIK3R2	RNF43	STAT5B	TTF1	PDGFB
APC	BUB1B	CSF1R	EP300	FH	ID3	MAP2K	NF2	PLK1	ROS1	STIL	TUBB3	RAF1
AR	C11orf30	CSF3R	EPCAM	FIP1L1	IDH1	MAP2K	NFKBIA	PMS1	RPTOR	STK11	TYMS	RARA
ARAF	CALR	CTCF	EPHA3	FLCN	IDH2	MAP2K	NKX2-1	PMS2	RRM1	STMN1	U2AF1	RET
ARID1A	CBL	CTLA4	ERBB2	FLT1	IGF1R	MAP3K	NOTCH1	POLD1	RUNX1	STX11	UGT1A	ROS1
ARID2	CCND1	CTNNB1	ERBB3	FLT3	IGF2	MCL1	NOTCH2	POLE	SBDS	STXBP2	UNC13	TMPRSS
ARID5B	CCNE1	CUX1	ERBB4	FLT4	IKBKE	MDM2	NPM1	POT1	SDHA	SUFU	VEGFA	
ASXL1	CCT6B	CXCR4	ERCC1	GADD45	IKZF1	MDM4	NQO1	PPP2R1A	SDHB	SUZ12	VHL	
ATM	CD22	CYLD	ERCC2	GATA1	IKZF2	MECOM	NRAS	PRDM1	SDHC	TEK	WISP3	
ATR	CD274	CYP2B6*6	ERCC3	GATA2	IKZF3	MED12	NRG1	PRF1	SDHD	TEKT4	WRN	
ATRX	CD58	CYP2C19*	ERCC4	GATA3	IL7R	MEF2B	NSD1	PRKAR1	SERP2	TERC	WT1	
AURKA	CD70	CYP2C9*3	ERCC5	GATA4	INPP4B	MEN1	NT5C2	PRKCI	SETBP1	TERT	XIAP	
AURKB	CDA	CYP2D6	ESR1	GATA6	INPP5D	MET	NTRK1	PTCH1	SETD2	TET2	XPC	
AXIN1	CDC73	CYP2D6*3	ETV1	GNA11	IRF1	MGMT	PAG1	PTEN	SF3B1	TGFBR2	XPO1	
AXL	CDH1	CYP2D6*4	ETV4	GNA13	IRF2	MITF	PAK3	PTPN11	SGK1	TLE1	XRCC1	
B2M	CDK10	CYP2D6*6	EWSR1	GNAQ	IRF8	MLH1	PALB2	PTPN2	SH2D1A	TLE4	YAP1	
BAP1	CDK12	CYP3A4*4	EZH2	GNAS	JAK1	MLL	PARK2	PTPN6	SMAD2	TMPRSS2	ZAP70	
BARD1	CDK4	CYP3A5*3	FANCA	GRIN2A	JAK2	MLLT10	PAX5	PTPRO	SMAD3	TNFAIP3	ZNF217	
BCL2	CDK6	DAXX	FANCB	GRM3	JAK3	MPL	PBRM1	QKI	SMAD4	TNFRSF1	ZNF703	
BCL2L1	CDK8	DDR2	FANCC	GSTM1	JARID2	MRE11	PC	RAC1	SMAD7	TNFRSF1	ZRSR2	
BCL2L2	CDKN1	DHFR	FANCD2	GSTP1	JUN	MSH2	PDCD1	RAD21	SMARCA4	TNFRSF1		
BCORL	CDKN1	DICER1	FANCE	GSTT1	KDM2	MSH3	PDCD1LG	RAD50	SMARCB1	TOP1		

Grey background indicates genes targeted for fusion detection

Supplementary Table 3. The total and common mutations identified in ctDNA and matched tumor samples

Patient No.	Mutations in Tumor	Mutations in ctDNA	Shared mutations	Mean coverage depth of ctDNA
1	2	2	2	558x
2	4	3	3	458x
3	2	3	2	562x
4	2	1	1	437x
5	3	8	3	403x
6	5	5	5	497x
7	3	3	3	635x
8	5	3	2	327x
9	3	2	2	389x
10	4	4	4	549x
11	2	2	2	412x
12	2	7	2	431x
13	7	8	7	416x
14	2	1	1	396x
15	4	4	4	426x
16	0	1	0	350x
17	3	1	1	579x
18	4	4	4	408x
19	2	2	2	336x
20	3	3	3	531x
21	2	5	2	342x
22	2	3	1	568x
23	3	3	3	548x
24	4	4	4	309x
25	3	3	2	353x
26	3	2	2	399x
27	1	1	1	369x
28	1	2	0	369x
29	3	4	2	457x
30	3	3	1	404x
31	5	3	3	416x
32	5	4	4	367x
33	2	2	2	362x
34	3	3	3	353x
35	1	1	1	330x
36	5	5	5	350x
37	3	3	3	302x
38	3	2	2	305x

39	4	4	4	376x
40	3	3	3	613x
41	3	3	3	330x
42	9	9	9	346x
43	2	4	2	307x
44	3	3	2	333x
45	3	4	3	454x
46	1	1	1	661x
47	3	4	3	351x
48	1	3	1	406x
49	4	4	4	337x
50	3	2	2	380x
51	3	5	3	505x
52	4	5	4	438x
53	2	3	2	467x
54	4	4	2	510x
55	11	10	10	519x
56	7	7	7	356x
57	5	5	5	495x
58	5	5	5	312x
59	3	3	3	303x
60	4	4	4	339x
61	16	16	16	419x
62	7	7	7	3064x
63	3	5	3	1315x
64	1	1	1	2048x
65	3	3	3	2979x
66	11	11	11	1771x
67	1	2	1	3911x
68	4	4	4	2116x
69	2	2	2	3371x
70	4	6	4	3867x
71	2	2	2	4318x
72	3	3	3	2320x
73	0	2	0	2858x
74	3	3	3	1657x
75	3	2	2	3852x
76	3	3	3	1966x
77	2	3	2	4336x
78	3	3	3	2324x
79	0	2	0	1021x
80	6	6	5	2711x

81	7	4	4	2257x
82	2	2	2	2248x
83	5	4	3	1985x
84	4	4	4	3167x
85	4	4	4	945x
86	2	4	1	887x
87	10	10	10	1799x
88	1	3	1	362x
89	3	2	2	2269x
90	2	2	2	977x
91	4	4	4	2257x
92	3	5	3	2950x
93	4	5	4	2362x
94	2	2	2	1593x
95	4	4	4	2778x
96	2	2	2	2706x
97	3	3	3	2677x
98	1	1	1	3273x
99	2	1	1	2508x
100	4	3	2	2458x
101	3	3	3	2936x
102	3	3	3	3499x
103	1	2	0	1606x
104	3	2	2	2664x
105	5	3	2	2078x
106	3	3	3	2495x
107	5	5	5	2276x
108	6	6	6	2523x
109	2	2	2	1919x
110	4	4	4	1979x
111	3	3	3	3020x
112	0	2	0	2212x
113	1	3	1	1965x
114	2	3	1	2166x
115	4	4	4	2385x
116	4	5	4	2439x
117	3	3	3	3019x
118	2	4	2	2362x
119	4	4	4	2864x
120	3	3	3	3100x
121	5	6	5	2337x
122	3	3	3	1549x

123	4	3	3	365x
124	2	2	2	392x
125	3	5	3	339x
126	5	3	3	337x
127	6	6	6	335x
128	2	2	2	302x
129	6	7	6	376x
130	2	1	1	327x
131	4	4	4	1950x
132	7	4	4	2688x
133	7	3	3	1816x
134	4	4	4	1704x
135	2	4	2	2016x
136	3	2	2	2072x
137	0	1	0	1824x
138	3	3	3	2047x
139	9	9	9	1850x
140	3	2	2	1146x
141	3	2	2	2101x
142	3	3	3	1787x
143	4	4	4	1850x
144	2	2	2	2093x
145	6	6	6	1964x
146	3	3	3	2091x
147	4	4	4	932x
148	3	3	3	2198x
149	1	1	0	1184x
150	1	1	1	2219x
151	2	3	0	2284x
152	6	6	6	1202x
153	2	3	2	2466x
154	0	1	0	2132x
155	2	2	2	2407x
156	3	3	3	2178x
157	3	2	2	1065x
158	5	2	2	1625x
159	1	1	1	1896x
160	2	1	1	1718x
161	2	2	2	1735x
162	4	4	4	1931x
163	3	3	3	2159x
164	3	3	3	946x

165	4	3	3	1887x
166	1	3	0	2505x
167	0	3	0	2070x
168	3	2	2	1908x
169	2	2	2	1939x
170	2	4	2	1980x
171	2	2	2	1826x
172	1	1	1	2052x
173	3	3	3	2154x
174	3	3	3	1243x
175	1	1	1	1145x
176	2	2	2	1118x
177	1	1	1	1200x
178	3	3	3	707x
179	4	4	4	1174x
180	4	4	4	1117x
181	2	5	2	965x
182	3	1	1	2139x
183	0	1	0	2014x
184	4	3	3	2070x
185	5	5	4	1493x
186	1	1	1	2535x
187	3	3	3	1914x
188	9	14	9	2213x
189	29	25	25	2283x
190	5	5	5	3000x
191	3	5	2	1511x
192	4	3	3	1605x
193	3	3	3	2131x
194	1	1	0	1956x
195	2	1	1	1775x
196	4	3	3	1475x
197	2	1	1	1268x
198	1	7	1	1901x
199	3	3	3	1298x
200	2	4	2	1705x
201	3	3	3	1655x
202	5	5	5	1878x
203	6	5	5	2092x
204	3	3	1	1436x
205	1	4	0	1852x
206	3	3	3	1485x

207	3	3	2	1891x
208	4	5	4	1298x
209	3	3	3	2466x
210	3	3	3	1943x
211	7	7	7	1777x
212	5	8	4	1524x
213	2	1	1	1979x
214	2	2	2	2178x
215	5	5	5	2025x
216	5	5	5	2517x
217	4	5	4	1861x
218	2	2	2	1760x
219	2	1	1	1091x
220	3	3	3	2025x
221	2	2	2	1889x
222	5	5	4	1532x
223	4	5	4	1480x
224	2	3	2	2156x
225	3	4	3	734x
226	6	5	5	2109x
227	4	3	3	2266x
228	3	1	1	1451x
229	2	2	2	2175x
230	3	3	3	1756x
231	4	4	4	2533x
232	0	2	0	2189x
233	4	4	4	1326x
234	3	3	3	1618x
235	4	3	3	2189x
236	3	3	3	2606x
237	3	2	2	2189x
238	8	8	8	2200x
239	3	3	3	1605x
240	3	3	2	1359x
241	6	7	6	1972x
242	4	5	4	1988x
243	2	3	2	1849x
244	4	4	4	2007x
245	3	4	3	1872x
246	2	1	1	2023x
247	2	2	2	1899x
248	0	2	0	1812x

249	8	8	8	2034x
250	7	7	7	2006x
251	8	8	8	2245x
252	7	7	7	2268x
253	1	2	1	1442x
254	1	1	1	1591x
255	3	3	3	1529x
256	2	2	2	1926x
257	2	2	2	1440x
258	3	3	3	2128x
259	13	6	6	1826x
260	1	1	1	1744x
261	2	2	1	1530x
262	4	4	4	1895x
263	3	2	2	1951x
264	0	2	0	1759x
265	1	2	0	2140x
266	7	6	6	1901x
267	3	4	3	1655x
268	1	3	1	1744x
269	2	2	2	2013x
270	2	2	2	2125x
271	4	1	1	1744x
272	5	5	5	1610x
273	4	4	4	2006x
274	6	6	6	1909x
275	4	2	2	2002x
276	5	4	4	1837x
277	6	4	4	1137x
278	8	11	6	2327x
279	2	2	2	2175x
280	2	2	2	3469x
281	6	6	6	1875x
282	3	3	3	2618x
283	4	2	1	361x
284	8	4	4	2533x
285	2	1	1	390x
286	2	1	1	1142x
287	3	1	1	2079x
288	2	1	1	2360x
289	11	11	11	403x
290	6	2	2	2212x

291	4	4	4	355x
292	2	0	0	301x
293	6	1	1	319x
294	3	1	1	333x
295	1	0	0	349x
296	3	0	0	359x
297	4	1	0	370x
298	2	1	1	392x
299	1	0	0	401x
300	3	0	0	491x
301	3	0	0	510x
302	2	1	0	808x
303	2	0	0	870x
304	2	0	0	895x
305	1	0	0	1302x
306	10	1	1	1440x
307	1	1	0	1447x
308	3	1	1	1477x
309	2	0	0	1489x
310	4	0	0	1512x
311	3	0	0	1527x
312	3	0	0	1561x
313	4	0	0	1574x
314	11	1	1	1581x
315	0	0	0	1699x
316	3	0	0	1727x
317	2	0	0	1737x
318	3	2	1	1747x
319	0	0	0	1788x
320	4	0	0	1797x
321	0	0	0	1845x
322	4	0	0	1911x
323	2	0	0	1925x
324	1	0	0	1997x
325	2	0	0	2006x
326	1	0	0	2013x
327	2	0	0	2060x
328	4	0	0	2063x
329	3	0	0	2117x
330	1	1	0	2118x
331	0	0	0	2160x
332	1	0	0	2190x

333	2	0	0	2204x
334	4	1	1	2206x
335	1	0	0	2328x
336	2	0	0	2375x
337	3	0	0	2387x
338	3	0	0	2465x
339	4	0	0	2523x
340	4	1	0	2728x
341	2	0	0	2849x
342	1	0	0	3104x
343	0	1	0	3281x
344	1	0	0	3445x

Supplementary Table 4. Potential clinical implications of actionable mutations

Gene	Mutations	Function	Clinical implications
ALK	Gene fusion	Gain	Decreased sensitivity to EGFR tyrosine kinase inhibitors (TKI) and increased sensitivity to ALK inhibitors.
AKT2	Gene amplification	Gain	Exert resistance to paclitaxel reagents.
BRAF	Missense mutation	Gain	Increased sensitivity to BRAF and MEK inhibitors.
BRCA1	Missense mutation	Loss	Decreased sensitivity to taxane-based (anti-microtubule) treatment; Increased sensitivity to platinum and mitomycin chemotherapy.
BRCA2	Nonsense mutation	Loss	Decreased sensitivity to taxane-based (anti-microtubule) treatment; Increased sensitivity to platinum and mitomycin chemotherapy.
CCND1	Gene amplification	Gain	Increased sensitivity to CDK4/6 inhibitors.
CCNE1	Gene amplification	Gain	May confer resistance to CDK4/6 inhibitors, such as palbociclib.
CDK4	Gene amplification	Gain	Increased sensitivity to CDK4/6 inhibitors.
CDK6	Gene amplification	Gain	Increased sensitivity to CDK4/6 inhibitors.
CDK12	Gene amplification	Gain	Possibly decreased sensitivity to PARP inhibitors.
DDR2	Gene amplification	Gain	Possible response to dasatinib or a combination of tyrosine kinase inhibitors
EGFR	Missense mutations; Gene amplification	Gain	Increased sensitivity to EGFR TKIs and anti-EGFR monoclonal antibodies.
	Missense mutation T790M	Gain	Sterically inhibit TKI binding; Increased sensitivity to third generation EGFR TKIs; decreased sensitivity to first and second generation EGFR TKIs.
ERBB2	Gene amplification	Gain	Increased sensitivity to second generation EGFR TKIs and anti-ERBB2 monoclonal antibodies.
FGFR1	Gene amplification	Gain	Increased sensitivity to FGFR kinase inhibitors.
FGFR4	Gene amplification	Gain	Increased sensitivity to FGFR kinase inhibitors.
FLT1	Gene amplification	Gain	Increased sensitivity to VEGFR kinase inhibitors.
FLT3	Gene amplification	Gain	Increased sensitivity to multi-TK inhibitors.
FLT4	Gene amplification	Gain	Increased sensitivity to VEGFR inhibitors.
HRAS	Missense mutations	Gain	Possibly increased sensitivity to MEK inhibitors.
KIT	Missense mutations	Gain	Increased sensitivity to several TKIs, such as imatinib and nilotinib
KRAS	Missense mutations	Gain	Possible acquired mutations during BRAF TKI treatment; Confers resistance to first generation EGFR TKIs; ERK inhibitors can block its downstream signals.
MAP2K2	Gene amplification	Gain	Increased sensitivity to MEK inhibitors.
MED12	Nonsense mutation	Loss	May confer resistance to ALK and EGFR inhibitors

MET	Gene amplification; Missense mutations	Gain	Sterically inhibit TKI binding; Confers resistance to EGFR TKIs; Increased sensitivity to MET inhibitors.
MTOR	Gene amplification	Gain	Increased sensitivity to mTOR inhibitors.
NF1	Frame shift and nonsense mutations	Loss	Possibly decreased sensitivity to BRAF inhibitors; Possibly ncreased sensitivity to MET inhibitors.
NF2	Nonsense mutation	Loss	Possibly increased sensitivity to EGFR and mTOR inhibitors.
NRAS	Missense mutation	Gain	Decreased sensitivity to first generation TKIs; ERK inhibitors may block its downstream signals.
PDGFRA	Gene amplification	Gain	Increased sensitivity to multi-TK inhibitors.
PDGFRB	Gene amplification	Gain	Increased sensitivity to multi-TK inhibitors.
PIK3CA	Missense mutation	Gain	Confers resistance to EGFR inhibitors; Increased sensitivity to PI3K/AKT/mTOR inhibitors.
PTEN	Nonsense mutation and frame shift	Loss	Decreased sensitivity to first generation EGFR TKIs; increased sensitivity to PI3K/AKT/mTOR inhibitors.
RB1	Nonsense mutation and frame shift	Loss	Increased sensitivity to genotoxic drugs; Decreased sensitivity to CDK4/CDK6 inhibitors.
RET	Gene fusion	Gain	Increased sensitivity to multi-TK inhibitors.
ROS1	Gene fusion	Gain	Increased sensitivity to ROS1 inhibitors, such as crizotinib; Decreased sensitivity to EGFR kinase inhibitors.
SMO	Gene amplification	Gain	Increased sensitivity to SMO antagonist.
TOP2A	Nonsense mutation	Loss	Decreased sensitivity to anthracycline.
TP53	Missense, nonsense mutation and frame shift	Loss	Decreased sensitivity to 5-fluorouracil and platinum based agents**.**
TSC1	Nonsense mutation and frame shift	Loss	Increased sensitivity to mTOR inhibitors.
TSC2	Nonsense mutation and frame shift	Loss	Increased sensitivity to mTOR inhibitors.
